# Revisiting postsurgical aortic pseudoaneurysm mortality and treatment options

**DOI:** 10.1007/s12471-024-01867-7

**Published:** 2024-04-03

**Authors:** Bert Bervoets

**Affiliations:** Dordrecht, The Netherlands

I read the study by Hegeman et al. [1] on transcatheter closure of postsurgical aortic pseudoaneurysms (PTAPs) with great interest but found two significant oversights in their work. Firstly, Matsuzawa et al. [2] did not report a 61% mortality rate in untreated PTAPs. In the same issue of the *Netherlands Heart Journal*, de Winter et al. [3] correctly identified Mulder et al. [4] as the source. However, Mulder’s study focuses on false aneurysms in the aortoiliac or aortofemoral region, not the ascending aorta, where the PTAPs of Hegeman et al. are located. Furthermore, Mulder’s reported 61% mortality rate from ruptures is actually only 44% (8 of 18 patients), a significant discrepancy.

Secondly, Hegeman et al. overlooked two vital treatment options: percutaneous transthoracic treatment [5] and a conservative approach for asymptomatic patients [6]. This omission is crucial, as 2 of 11 patients in their study died post-treatment. Incorporating these methods could potentially offer safer alternatives and improve patient outcomes.
